# A database on the abundance of environmental antibiotic resistance genes

**DOI:** 10.1038/s41597-024-03084-8

**Published:** 2024-02-27

**Authors:** Wenjuan Xu, Zhizhen Pan, Yangyu Wu, Xin-Li An, Weiyi Wang, Boris Adamovich, Yong-Guan Zhu, Jian-Qiang Su, Qiansheng Huang

**Affiliations:** 1 Xiamen Key Laboratory of Indoor Air and Health, Key Lab of Urban Environment and Health, Institute of Urban Environment, Chinese Academy of Sciences, Xiamen, 361021 China; 2 College of Resources and Environment, University of Chinese Academy of Sciences, Beijing, 100049 China; 3 College of Life Sciences, Institute of Life Sciences and Green Development, Hebei University, Baoding, 071002 China; 4 Research Laboratory of Aquatic Ecology, Belarusian State University, Minsk, 220030 Belarus; 5 National Basic Science Data Center, Beijing, 100190 China

**Keywords:** Computational biology and bioinformatics, Environmental monitoring

## Abstract

Antimicrobial resistance (AMR) poses a severe threat to global health. The wide distribution of environmental antibiotic resistance genes (ARGs), which can be transferred between microbiota, especially clinical pathogens and human commensals, contributed significantly to AMR. However, few databases on the spatiotemporal distribution, abundance, and health risk of ARGs from multiple environments have been developed, especially on the absolute level. In this study, we compiled the ARG occurrence data generated by a high-throughput quantitative PCR platform from 1,403 samples in 653 sampling sites across 18 provinces in China. The database possessed 291,870 records from five types of habitats on the abundance of 290 ARGs, as well as 8,057 records on the abundance of 30 mobile genetic elements (MGEs) from 2013 to 2020. These ARGs conferred resistance to major common types of antibiotics (a total of 15 types) and represented five major resistance mechanisms, as well as four risk ranks. The database can provide information for studies on the dynamics of ARGs and is useful for the health risk assessment of AMR.

## Background & Summary

Antimicrobial resistance (AMR) has been recognized as a serious threat to global public health^1,2^. A global study published in *The Lancet* showed that AMR contributed directly to about 1.27 million deaths in 2019^3^. A report entrusted by the UK Government estimated that AMR may carry off 10 million people each year by 2050^4^. The increasing AMR risk is largely attributed to the spread of antibiotic resistance genes (ARGs), which bacteria can acquire to develop resistance to antibiotics^5,6^. ARGs present in the environment can be horizontally and vertically transferred within the microbial community, thus facilitating the spread of AMR^6–8^. ARGs confer resistance to a variety of antibiotics, including aminoglycoside, tetracycline, macrolide, chloramphenicol, glycopeptide, sulfonamide, trimethoprim, beta-lactams, fluoroquinolone, quinolones and so on^9,10^. Six major resistance mechanisms were found in the antibiotic mechanism of ARGs, including reducing intracellular antibiotic concentrations by efflux pumps, inactivating the antibiotics by hydrolysis or modification, altering, replacing, or protecting the antibiotic target, as well as cellular protection with poor permeability of antibiotics^11–13^.

Actually, ARGs have already existed in environmental bacteria for millions of years, predating the discovery and use of antibiotics. The presence of several ARGs were found from the ancient layers of the Canadian high Arctic permafrost^14^. Anthropogenic activities, particularly the overuse and misuse of antibiotics in agriculture, human medicine, and animal husbandry, have accelerated the enrichment and dissemination of ARGs^15–18^. As emerging contaminants, ARGs have been globally detected in a diversity of environmental compartments, including water, soil, and air^19–27^. The abundance of ARGs varied across different regions, whereas hotspots are found in antibiotic-enriched areas, including hospitals, antibiotic wastewater and farms^16,28,29^. It has been confirmed that the ARG abundance increased with the anthropogenic activity^30^. With the aid of mobile genetic elements (MGEs), ARGs can be horizontally transferred from environmental antibiotic-resistant bacteria to clinical pathogens, further exacerbating the risks to human health^30,31^. Consequently, the risk assessment of ARGs has become a global urgence, where the primary step is to reveal the occurrence, distribution, and abundance of ARGs in the environment.

Currently, metagenomic analysis and quantitative PCR are two popular approaches for detecting the composition and abundance of ARGs^28,32^. The profiles of ARGs can be predicted using metagenomic sequencing data annotated by blasting against the reference database, such as Antibiotic Resistance Genes Database^33^, the Comprehensive Antibiotic Research Database^34^ and the Structured Antibiotic Resistance Gene^35^. For instance, Zheng, *et al*.^36^ generated a global abundance map of 558 ARGs in the soil using metagenomic sequencing. Hendriksen, *et al*.^37^ use metagenomic analysis of sewage in 60 countries for the prediction of AMR. This method has the capacity to provide a comprehensive overview of ARG profiles in a specific sample. In contrast, quantitative PCR, especially high-throughput quantitative PCR (HT-qPCR), showed better detection limits, lower cost, reduced sample quantity requirement, and the ability for absolute quantification^38,39^. Willms, *et al*.^40^ utilized real-time quantitative PCR to quantify medically relevant ARGs and MGEs in the soil in Germany, and Delgado-Baquerizo, *et al*.^41^ generated a global atlas of soil ARG abundance detected by HT-qPCR. The HT-qPCR method was established in our lab and widely used^22,42,43^. With this technology, large amounts of data about the levels of ARGs in the environment were produced.

To the best of our knowledge, there are few databases gathering comprehensive information on the distribution and abundance of ARGs from various environments in a time- and space-dependent way, especially regarding the absolute levels of ARGs. In this study, a database was provided on the occurrence of environmental ARGs through HT-qPCR in China from 2013 to 2020. In detail, the data on the relative and absolute abundance of ARGs and MGEs from five types of habitats (aquatic, edaphic, sedimentary, dusty, and atmospheric environments) were presented in a spatiotemporal way. This database can serve as a valuable resource to provide fundamental data for facilitating the understanding of the diversity and abundance of environmental ARGs, as well as for evaluating the health risk of ARGs.

## Methods

### Sample collection

The soil samples were collected from the top 20 cm of soil by taking, gathering, and mixing several soil cores at each sampling site after plant residue and stones had been removed^43^. Water samples were aseptically taken from 10 to 20 cm below the water surface using sterilized containers^44,45^. Sediment samples were collected from the top 15 cm of sediment and stored in well-sealed and sterile plastic bags^22^. Particulate matter (PM_2.5_ and PM_10_) samples were pooled using the portable atmospheric particulate matter samplers with PM_2.5_ and PM_10_ fractionating inlets, respectively, and the samples were enriched with quartz microfiber filters^46^. These PM samples were described as being collected from the air habitat in this study. Dust was collected from outdoor and indoor settings with a clean sterile brush^46^. All the samples were promptly frozen, transported to the laboratory, and stored at 4 °C or −20 °C until next process.

### Determination of gene abundance

The DNA was extracted from samples using commercial DNA extraction Kit following the manufacturer’s instructions. Then the abundance of ARGs and MGEs were both analysed by HT-qPCR with the SmartChip Real-time PCR system (Warfergen Inc. USA)^22,42^. A total of 414 primer pairs were used to target 290 subtypes of ARGs, 16 transposases, 6 plasmids, 5 insertion sequences, 3 integrases and the 16S rRNA gene (Table S1). To verify the specificity of each primer set, a non-template negative control was performed. The thermal cycle consisted of an initial denaturation at 95 °C for 10 min, followed by 40 cycles of denaturation at 95 °C for 30 s and annealing at 60 °C for 30 s, finally with a melting curve analysis. The PCR reaction was conducted in triplicate for each primer set for each sample. The detection limit was set up at the threshold cycle (*Ct*) lower than 31. If the value of *Ct* exceeded 31, the corresponding data about the gene abundance would be regarded as 0. For each sample, only data with more than two repeated technical replicates above the detection limit were regarded as positive^47^. The gene copy number was calculated by Eq. (1), and the relative abundance of each gene represented the ratio of its copy number to 16S rRNA gene copy number (Eq. (2))^48^. The level of 16S rRNA was widely used to show the abundance of bacteria. Its absolute copy number was determined by plotting a standard curve using the Roche 480 system. The standard curve was obtained by a series of 10-fold dilutions 10-fold dilution of a standard plasmid containing a cloned and sequenced 16S rRNA gene fragment (1.39 × 10^10^ copies/L). The PCR reaction was run in triplicate with negative and positive controls. The absolute abundance of ARGs was calculated by multiplying with 16S rRNA gene absolute copy number as shown in Eq. (3)^47^.

1 $${\rm{G}}{\rm{e}}{\rm{n}}{\rm{e}}\,{\rm{c}}{\rm{o}}{\rm{p}}{\rm{y}}\,{\rm{n}}{\rm{u}}{\rm{m}}{\rm{b}}{\rm{e}}{\rm{r}}={10}^{(31-{\rm{C}}{\rm{t}})/(10/3)}$$

2 $${\rm{Gene}}\;{\rm{relative}}\;{\rm{abundance}}=\frac{{\rm{Gene}}\;{\rm{copy}}\;{\rm{number}}}{{\rm{16S}}\;{\rm{rRNA}}\;{\rm{gene}}\;{\rm{copy}}\;{\rm{number}}}$$

3 $${\rm{Gene}}\,{\rm{absolute}}\,{\rm{abundance}}={\rm{Gene}}\,{\rm{relative}}\,{\rm{abundance}}\times 16{\rm{S}}\,{\rm{rRNA}}\,{\rm{gene}}\,{\rm{absolute}}\,{\rm{copies}}$$

### Data processing

**Fig. 1:**
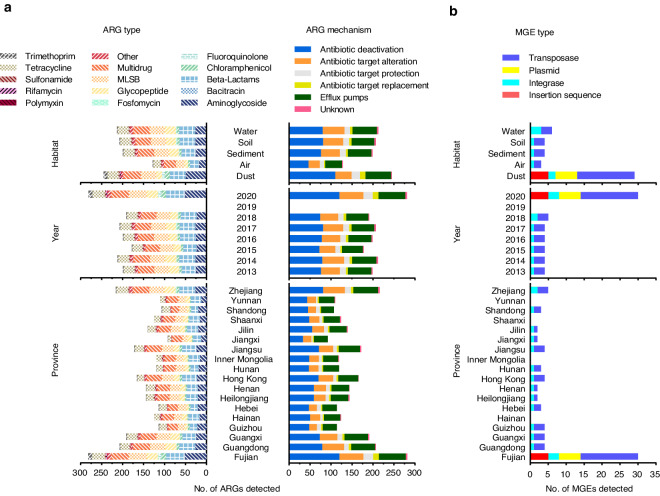
The number of ARGs (**a**) and MGEs (**b**) detected in each habitat, each year from 2013 to 2020 and each province of China. ARGs were classified based on antibiotics to which they conferred resistance and resistance mechanism, respectively.

The data were obtained from our and the collaborator’s laboratories. We reclassified the ARGs into 15 types according to the antibiotics type to which they conferred resistance, including aminoglycoside, beta-lactams, fluoroquinolone, glycopeptide, macrolide-lincosamide-streptogramin B (MLSB), sulfonamide, tetracycline, chloramphenicol, trimethoprim, multidrug, rifamycin, fosfomycin, bacitracin, polymyxin and others. Furthermore, all the genes were sorted into a total of 290 different subtypes. As shown in Fig. 1a, a total of 290 ARG subtypes were detected in environmental samples, and the number of ARGs detected in each environment ranged from 128 to 245, with an average of 198. Multidrug, MLSB, and beta-lactams resistance genes were the major types, followed by aminoglycoside, tetracycline, and glycopeptide resistance genes in each habitat. In terms of the time series, the number of ARGs detected from 2013 to 2018 and in 2020 ranged from 178 to 281, with an average of 183. The top six most detected types of ARGs (multidrug, MLSB, beta-lactams, tetracycline, aminoglycoside, and glycopeptide resistance genes) every year were the same in every habitat. Regarding the spatial scale, the number of ARGs detected in each province ranged from 92 to 282, with an average of 149. The dominant types of ARGs in each province were the same as mentioned above. In addition, a total of 30 MGEs (5 insertion sequences, 6 plasmids, 3 integrase genes, and 16 transposase genes) were detected and the highest number of MGEs was detected in the dust samples with all four types detectable. In the air, sediment, and water samples, integrase genes and transposase genes were detected. In a temporal scale, the highest number of detected MGEs occurred in 2020, encompassing all four types, whereas only integrase genes and transposase genes were detected in the other years. In terms of spatial distribution, the highest number of detected MGEs was in Fujian, and all four types were involved, while only two types, integrase genes and transposase genes, were detected in other provinces (Fig. 1b). If the genes detected in the same sample belonged to the same subtype, we would regard the sum of their abundance data as the abundance of that subtype. For example, the sum of the abundance of *acrA-01*, *acrA-02*, *acrA-03*, *acrA-04*, and *acrA-05* is regarded as the abundance of *acrA*.

**Fig. 2:**
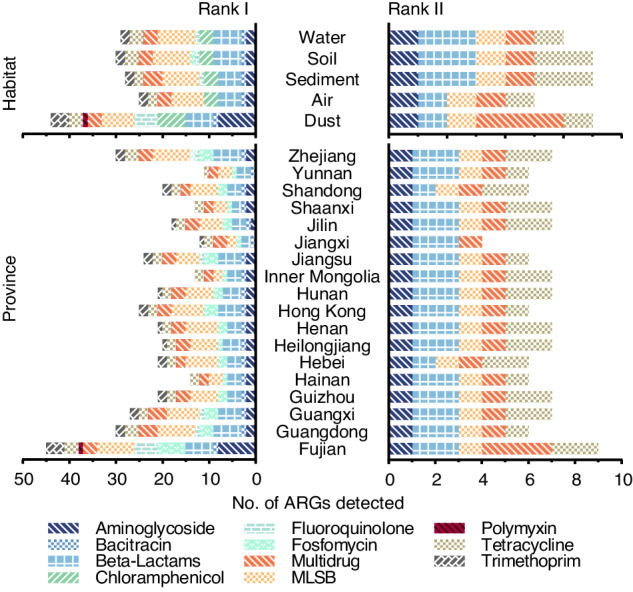
The number of detected risk health Rank I and II ARGs. ARGs were classified based on antibiotics to which they conferred resistance.

The ARGs were also divided into six groups based on the mechanism of resistance: antibiotic deactivation, efflux pumps, antibiotic target alteration, antibiotic target protection, antibiotic target replacement, and unknown. Antibiotic deactivation, antibiotic target alteration, and efflux pumps were the three most dominant resistance mechanisms (Fig. 1a). In addition, the health risk of ARGs was categorized into four ranks according to a previous study^49^. Rank I, as the highest risk, refers to mobile ARGs that are present in ESKAPE (*Enterococcus faecium*, *Staphylococcus aureus*, *Klebsiella pneumoniae*, *Acinetobacter baumannii*, *Pseudomonas aeruginosa*, and *Enterobacter* species) pathogens. Rank II indicates mobile ARGs that are not yet present in pathogens. Rank III reflects ARGs that are human-associated but not associated with MGEs, while Rank IV, as the lowest risk, denotes ARGs that are not human-associated. We identified the health risk rank of ARGs based on the subtype. When a subtype possessed more than one rank, the highest risk rank would be assigned to this subtype. In this database, among all the ARGs, 47 subtypes were identified as Rank I ARGs, 9 as Rank II ARGs, 53 as Rank III ARGs, and 79 as Rank IV ARGs. Rank I and Rank II ARGs, as the current and future threats, respectively, deserve public attention. The number of the Rank I ARGs detected in each habitat ranged from 25 to 44. They consisted of 11 types of ARGs, with MLSB and beta-lactams resistance genes being the top two types. For Rank II ARGs, 5 to 7 genes belonging to five types were detected in each environment. Among 18 provinces, the number of detected Rank I ARGs ranged from 11 to 45, and the top two dominant ARGs were MLSB and beta-lactams resistance genes. The number of detected Rank II ARGs ranged from 4 to 9 (Fig. 2).

## Data Records

The data can be downloaded in the form of Excel spreadsheets (.xlsx) from 10.57760/sciencedb.09803^50^ and the online platform is available at http://sdc.iue.ac.cn/abundance/arg/list.

The database consists of two separate tables, providing the abundance of ARGs and MGEs with detailed gene information and sample information, respectively. Each gene was assigned a unique Gene ID. For gene information, the resistance type, resistance mechanism, and health risk rank were introduced to classify different ARG subtypes. The types of MGEs are also provided. Each sample has a unique sample ID, which in turn can be related to a source ID. The source ID can be linked to the information about the publications using the data in the database. The location ID was also introduced, allowing distinguishing samples from different sites. The information about the geographic location of samples comprises the country, province, longitude, and latitude. All the information that is necessary to identify and retrace a sample, including the IDs assigned by us, the original ID used in the original studies, sampling date, sampling sites, and habitat, is provided. An explanatory note can be downloaded together with the database. An online platform with a user-friendly interface has been developed and can provide search service to obtain profiles and abundance of ARGs in a specific period and location.

**Fig. 3:**
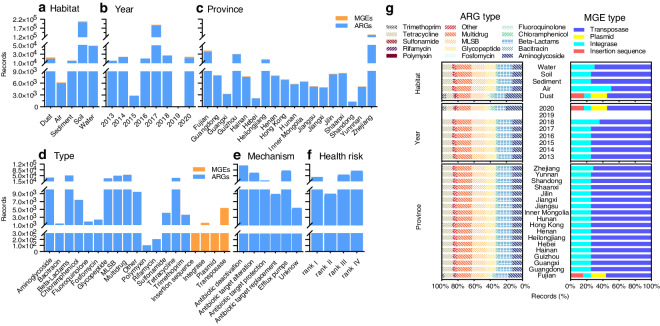
The abundance records of ARGs and MGEs in the database. The number of abundance records across habitats (**a**), years (2013−2020) (**b**), and provinces in China (**c**). The number of abundance records across ARG and MGE types (**d**), and ARG mechanisms (**e**). (**f**) The number of ARG abundance records across four health risk ranks. (**g**) The proportion of abundance records for ARG and MGE type in each habitat, each year and each province of China.

**Fig. 4:**
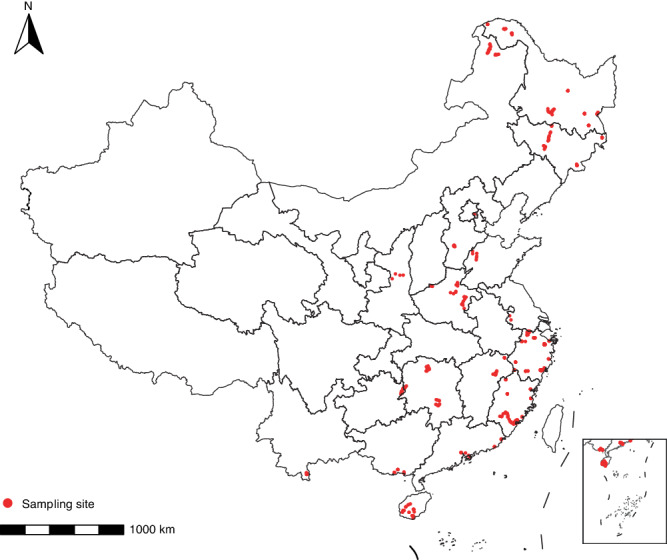
Map of sampling areas across China. The red dots indicate the sampling sites.

The database includes 291,870 records on ARGs, and 8,057 records on MGEs. For ARGs, there were 203,138 records from soil habitat, 48,354 records from water habitat, 19,476 records from dust habitat, 14,910 records from sediment habitat, and 5,992 records from air habitat. For MGEs, there were 3,844 records from soil habitat, 694 records from water samples, 3,071 records from dust habitat, 280 records from sediment habitat, and 168 records from air habitat (Fig. 3a). As shown in Fig. 3b, the ARG abundance data for 2017 were the largest (n = 185,376), followed by 2018 (n = 26,750) and 2020 (n = 20,974), while the MGEs abundance data for 2017 were the largest (n = 3,512), followed by 2020 (n = 3,113) and 2018 (n = 584). As shown in Fig. 3c and Fig. 4, there were 653 sampling sites across 18 provinces in China, mainly in the eastern region, which is regarded as the hotspot region of antibiotic pollution^51–53^. The top four provinces with the highest number of ARG abundance data were Zhejiang (n = 130,472), Fujian (n = 34,312), Guizhou (n = 29,610) and Heilongjiang (n = 17,430). For MGEs, abundance data in Fujian (n = 3,365) and Zhejiang (n = 2,276) were the largest, more than four times the number in other provinces (Fig. 3c). The ARG abundance records cover 15 types of ARGs. Among them, the multidrug (n = 54,542), beta-lactams (n = 50,588), MLSB resistance genes (n = 49,375), aminoglycoside (n = 37,508), tetracycline (n = 36,011), glycopeptide (n = 33,235) had the highest number of the records, and they were most studied in each habitat, each year or each province (Fig. 3d,g). Four types of MGEs were included. The number of abundance data about transposase genes, integrase genes, plasmids, and insertion sequences were 5,356, 1,568, 618, and 515, respectively, and transposase genes were most studied in each habitat, each year or each province (Fig. 3d,g). The ARGs contain six antibiotic mechanisms. Antibiotic deactivation (n = 112,698), efflux pumps (n = 79,248), and antibiotic target alteration (n = 66,614) had the major number of the abundance data (Fig. 3e). In addition, among four health risk ranks of ARGs, Rank IV ARGs (n = 78,303) had the largest number of the abundance data, followed by Rank III ARGs (n = 54,091) and Rank I ARGs (n = 42,287). Rank II ARGs (n = 8,911) had the lowest number of the abundance data (Fig. 3f).

## Technical Validation

All the data were uniformly generated using HT-qPCR under the same standard operational protocols, thus showing high comparability among samples. For each gene, at least two replicates producing the threshold cycle lower than 31 were obtained. With this technology, large amounts of data were produced. Approximately 48.63% of the data were applied to the research paper published in *Nature Microbiology*, *Environmental International*, and other journals^22,42–46,54,55^.

### Abundance of ARGs

**Fig. 5:**
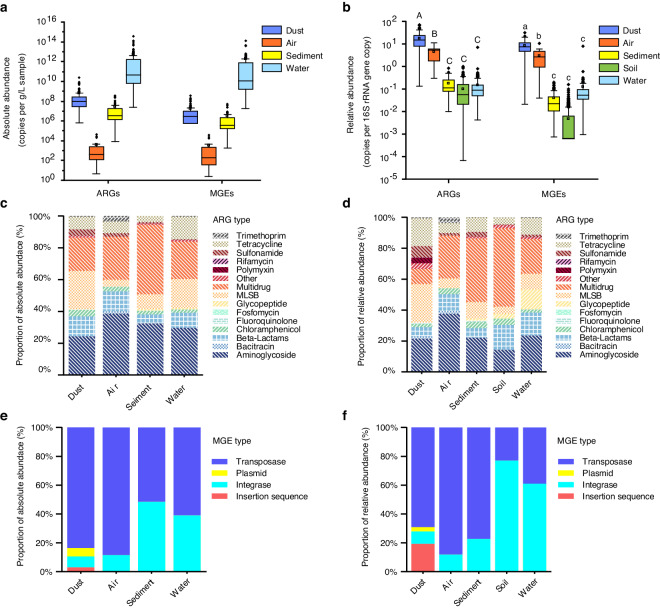
The abundance of ARGs and MGEs in various habitats. Absolute abundance (**a**) and relative abundance (**b**) of ARGs and MGEs in various habitats. The middle horizontal line and square represent the median and mean, the box denotes the 25% and 75% percentiles, the whiskers refer to the range within 1.5 × IQR (interquartile range), data beyond the whiskers are outlying points. Different letters in (**b**) indicate statistically significant differences among different habitats (*P* < 0.05, one-way ANOVA with Tukey’s test). Capital letters are for ARGs and lowercase letters are for MGEs. The proportion of absolute abundance (**c**) and relative abundance (**d**) of ARG types. The proportion of absolute abundance (**e**) and relative abundance (**f**) of MGE types.

The relative abundance and absolute abundance of ARGs are available for the five habitats in the database, except for soil which only the relative abundance data can be obtained. The abundance of ARGs in various habitats is shown in Fig. 5. The average absolute abundance of ARGs in dust, air, sediment, and water were 6.22 × 10^8^ copies/g, 4.11 × 10^3^ copies/L, 2.67 × 10^7^ copies/g, 5.61 × 10^12^ copies/L, respectively. The median absolute abundance of ARGs in dust, air, sediment, and water were 9.86 × 10^7^ copies/g, 3.92 × 10^2^ copies/L, 3.48 × 10^6^ copies/g, 4.54 × 10^10^ copies/L respectively (Fig. 5a). The proportion of absolute abundance of ARG types was different in each habitat. For example, in the sediment habitat, multidrug and aminoglycoside resistance genes accounted for 43.64% and 32.40%, respectively, while in dust habitat, aminoglycoside (24.27%), MLSB (24.18%) and multidrug resistance genes (20.87%) were the most abundant ARGs (Fig. 5c). To compare the relative abundance of ARGs among different habitats, one-way ANOVA with Tukey B post hoc analysis was conducted. The difference was considered statistically significant at a *P* value less than 0.05. The dust had the highest relative abundance of total ARGs (average = 18.00 copies per 16S rRNA gene copy, median = 15.02 copies per 16S rRNA gene copy), followed by atmospheric environment (average = 4.54 copies per 16S rRNA gene copy, median = 5.21 copies per 16S rRNA gene copy) (Fig. 5b). To be noted, the major dust samples were collected from the hospital, which could be the reason for relative high abundance of ARGs. The relative composition of different types of ARGs varied from one habitat to another. For example, genes conferring multidrug resistance (50.63%) dominated in the soil environment, while ARGs conferring resistance to aminoglycoside (37.62%) had a high proportion in the atmospheric environment (Fig. 5d).

**Fig. 6:**
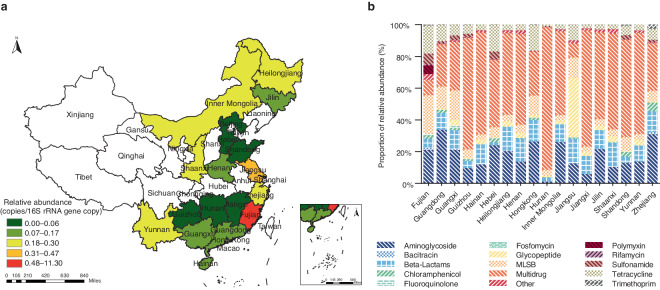
Distribution of ARGs in China. (**a**) Spatial distribution of total ARGs relative abundance in China. (**b**) Relative abundance of ARG types (%) in China.

**Fig. 7:**
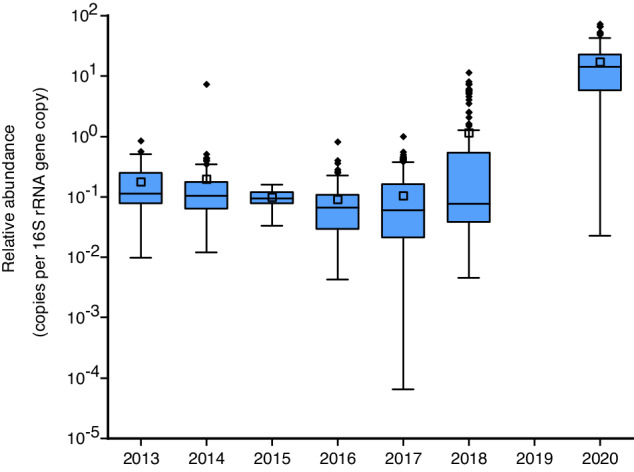
Relative abundance of total ARGs from 2013 to 2020. The middle horizontal line and square represent the median and mean, the box denotes the 25% and 75% percentiles, the whiskers refer to the range within 1.5 × IQR, data beyond the whiskers are outlying points.

As shown in Fig. 6a, the relative abundance of total ARGs ranged from 7.40 × 10^−3^ to 11.30 copies per 16S rRNA gene copy across 18 provinces in China. The map showed that the average relative abundance of ARGs in Fujian was the highest, 2–4 orders of magnitude higher than in other provinces. This may be because most of the dust samples were collected in Fujian. Among 18 provinces, Fujian, Jiangsu, Zhejiang, Heilongjiang, Shaanxi, Inner Mongolia, and Yunnan were the top seven provinces with the highest relative abundance of ARGs, while Shandong, Hebei, Hunan, Guizhou, Hongkong, and Jiangxi had the lowest abundance. For the relative composition of ARGs, as shown in Fig. 6b, in most provinces, the multidrug resistance genes were the dominant ARGs (above 42.41%), while the glycopeptide resistance genes were the major ARGs in Jiangsu (37.40%). Aminoglycoside and multidrug resistance genes dominated in Zhejiang, Hong Kong, and Guangdong (60.84%, 53.95%, and 60.22%, respectively). The genes conferring resistance to MLSB (24.37%), aminoglycoside (21.06%), and tetracycline (17.59%) were the top three ARGs in Fujian. Figure 7 displays the relative abundance of total ARGs from 2013 to 2020, ranging from 9.09 × 10^−2^ to 16.94 copies per 16S rRNA gene copy. The average relative abundance of ARGs in 2018 and 2020 was higher than in other years, to a great extent, due to the dust and air samples which were obtained in 2018 and 2020.

### Abundance of MGEs

Both the relative abundance and absolute abundance of MGEs are available for the five habitats in the database, except soil which only the relative abundance data can be obtained. The average absolute abundance in dust, air, sediment, and water were 2.01 × 10^8^ copies/g, 3.53 × 10^3^ copies/L, 3.35 × 10^6^ copies/g, 1.96 × 10^12^ copies/L, respectively. The median absolute abundance of MGEs in dust, air, sediment, and water were 4.43 × 10^7^ copies/g, 2.02 × 10^2^ copies/L, 3.82 × 10^5^ copies/g, 1.17 × 10^10^ copies/L, respectively (Fig. 5a). Transposase genes accounted for 83.62%, 88.51% and 61.00% of MGEs absolute abundance in dust, air and water, respectively. In sediment, transposase genes (51.62%) and integrase genes (48.38%) were the major abundant MGEs (Fig. 5e). Considering the relative abundance of MGEs, from our data, dust and air were the two habitats with the significant highest average relative abundance (8.25 and 3.00 copies per 16S rRNA gene copy respectively) (*P < *0.05, one-way ANOVA with Tukey B post hoc analysis), which were 1–3 orders of magnitude higher than other habitats (Fig. 5b). Transposase genes dominated in dust, air, and sediment (69.15%, 88.17% and 77.37%, respectively), while integrase genes accounted for a large proportion in soil and water (77.00% and 61.04%) (Fig. 5f).

## Usage Notes

The data are shared under Creative Commons Attribution 4.0 International (CC BY 4.0) and the license text can be available at https://creativecommons.org/licenses/by/4.0/. The data can be downloaded from Scientific Data Bank^50^, where users can also access when clicking ‘download’ on online platform (http://sdc.iue.ac.cn/abundance/arg/list).

The database provides a valuable resource for exploring the patterns of antibiotic resistance and studying the transfer of ARGs facilitated by MGEs. The spatial distribution analysis of ARGs can be used to identify microbial resistance hotspots. The database also provides basic information for studying the risk of antibiotic resistance.

### Supplementary information

Supplementary Table 1

## Data Availability

No custom code was made during the collection and validation of this dataset.
